# Insights into ageing rates comparison across tissues from recalibrating cerebellum DNA methylation clock

**DOI:** 10.1007/s11357-023-00871-w

**Published:** 2023-08-19

**Authors:** Yucheng Wang, Olivia A. Grant, Xiaojun Zhai, Klaus D. Mcdonald-Maier, Leonardo C. Schalkwyk

**Affiliations:** 1 School of Computer Science and Electronic Engineering, University of Essex, Colchester, CO4 3SQ UK; 2 School of Life Sciences, University of Essex, Colchester, CO4 3SQ UK; 3 Institute of Social and Economic Research, University of Essex, Colchester, CO4 3SQ UK; 4 Blizard Institute, Barts and The London School of Medicine and Dentistry, Queen Mary University of London, London, E1 2AT UK

**Keywords:** Ageing rate, Epigenetic clocks, Cerebellum, DNA methylation

## Abstract

DNA methylation (DNAm)-based age clocks have been studied extensively as a biomarker of human ageing and a risk factor for age-related diseases. Despite different tissues having vastly different rates of proliferation, it is still largely unknown whether they age at different rates. It was previously reported that the cerebellum ages slowly; however, this claim was drawn from a single clock using a relatively small sample size and so warrants further investigation. We collected the largest cerebellum DNAm dataset (*N* = 752) to date. We found the respective epigenetic ages are all severely underestimated by six representative DNAm age clocks, with the underestimation effects more pronounced in the four clocks whose training datasets do not include brain-related tissues. We identified 613 age-associated CpGs in the cerebellum, which accounts for only 14.5% of the number found in the middle temporal gyrus from the same population (*N* = 404). From the 613 cerebellum age-associated CpGs, we built a highly accurate age prediction model for the cerebellum named CerebellumClock_specific_ (Pearson correlation=0.941, MAD=3.18 years). Ageing rate comparisons based on the two tissue-specific clocks constructed on the 201 overlapping age-associated CpGs support the cerebellum has younger DNAm age. Nevertheless, we built BrainCortexClock to prove a single DNAm clock is able to unbiasedly estimate DNAm ages of both cerebellum and cerebral cortex, when they are adequately and equally represented in the training dataset. Comparing ageing rates across tissues using DNA methylation multi-tissue clocks is flawed. The large underestimation of age prediction for cerebellums by previous clocks mainly reflects the improper usage of these age clocks. There exist strong and consistent ageing effects on the cerebellar methylome, and we suggest the smaller number of age-associated CpG sites in cerebellum is largely attributed to its extremely low average cell replication rates.

**Supplementary Information:**

The online version contains supplementary material available at 10.1007/s11357-023-00871-w.

## Introduction

Ageing is characterized by progressive loss of cellular functions, leading to increased risk of morbidity and mortality [1]. A significant challenge in the ageing field is how to accurately measure ageing. Further investigation of ageing biomarkers will not only increase our knowledge of the mechanisms of ageing, but also facilitate monitoring the various interventions for improving human healthspan and rejuvenation experiments. In the past decades, a variety of ageing biomarkers, such as telomere attrition [2], DNA methylation (DNAm) changes [3, 4], and alterations in gene expression [5, 6] and metabolite concentration [7, 8], have attracted even more attention and were used to build age estimators or age clocks to measure the biological age [9]. Among them, age clocks based on DNAm changes, also called epigenetic clocks, were demonstrated to be the most accurate and robust age estimators; they are the most promising ageing biomarkers that can be applied to individuals [10]. Age-related DNAm changes are widespread across the genome, throughout the life course [3, 11–13] and exist in a wide variety of tissues [4, 14].

Since 2013, many DNAm-based age clocks have been published. Among them, Hannumn’s clock [3] and Horvath’s clock [4] are two first and most widely used DNAm clocks, especially Steve Horvath first demonstrated that a relatively accurate age prediction (median absolute error 3.6 years) is possible for multiple distinct tissues via a single linear model that includes only a small number of CpGs [4]. Different tissues may have distinct DNA methylation profiles; therefore, many tissue-specific clocks have been developed and demonstrated better age prediction performance than the multi-tissue clock; tissue-specific clocks have been developed for buccal cells [15], brain cortex [16], skin [17, 18], breast [19], and so on. Age acceleration, a popular concept that is defined as the difference between predicted DNAm age and the chronological age, derived from Hannnum’s clock or Horvath’s clock has been documented to be associated with a variety of age-related conditions and diseases [20–23]. However, Zhang et al. reported that the association between age acceleration and mortality decreased to non-significant with increased accuracy of chronological age prediction of age models [24]. A recent preprint manuscript reported that the attenuated biological associations are due to clocks trained on large-size sample sets having incorporated lifestyle-associated non-age-correlated CpGs [25]. Meanwhile, instead of directly regressing on the chronological age, two other DNAm clocks—PhenoAge [26] and GrimAge [27], which were regressed on estimated phenotypic age and mortality risk, respectively—were reported to better predict lifespan and healthspan than previous chronological age clocks.

Ageing is generally considered a gradual process that happens to the body as a whole. It is still an open question whether different organs/tissues have different ageing rates. Furthermore, how can we truthfully compare the ageing rates between different tissues? Horvath’s pan-tissue clock gives excellent accuracy in estimating DNAm age for many different cells and tissues [4], which may suggest that those different cells and tissue types may have similar ageing rates. In 2015, Horvath et al. claimed that the cerebellum ages slower than many other parts of the human body based on the observations that the DNAm age of the cerebellum is much lower than other tissues based on the pan-tissue clock [28]. In addition, Horvath and his colleagues also claimed that women’s breast tissues have a relatively higher DNAm ageing rate [4, 29]. If it is true that some tissues have significantly different DNAm ageing rates than other tissues, then we can go further to identify what drives the difference. This is a very important angle to understand the mechanisms of age-associated DNA methylation changes. Even though there have been reported many strong age-associated CpG sites, there is still very little known about the underlying mechanisms that drive age-associated DNA methylation changes [30–32].

In recent years, many more cerebellum DNA methylation samples have become publicly available, and many diverse DNAm age clocks have also been developed [33]. We set out to examine the claim that the cerebellum ages slowly within a much larger size dataset and find out the mechanisms. To achieve that, we first collect the largest cerebellum DNA methylation sample dataset, then compare their estimated epigenetic ages from six representative DNAm age clocks. After that, we perform age EWAS for cerebellums and middle temporal gyrus, separately on the same large-size elderly population (*n* = 404) to reveal the distinct age-associated methylomic changes of the cerebellum. Lastly, we construct cerebellum-specific clocks and further examine the claim that the cerebellum ages slower.

## Methods

### DNAm datasets

The DNAm samples were collected from the public data repository—Gene Expression Omnibus (GEO). The cerebellum samples are from six datasets, including GSE134379 [34], GSE59685 [35], GSE105109 [36], GSE125895 [37], GSE61431 [38], and GSE72778 [39]. They were included according to the following criteria: contain at least 20 cerebellum samples; with age annotations, and raw IDAT files or methylated and unmethylated intensity files are available. The cerebellum samples were used to reveal the underestimation issues for the cerebellum tissue by six representative clocks and were also used to train cerebellum age clocks. Apart from cerebellum samples, GSE134379 [34] also includes DNAm microarray data of the middle temporal gyrus from the same 404 individuals; thus, it was used to perform age EWASs on the two brain tissues. GSE59685 [35] includes 531 DNAm samples of five tissues, i.e., cerebellum, entorhinal cortex, frontal cortex, superior temporal gyrus, and whole blood, from donors (*N* = 122) archived in the MRC London Brainbank for Neurodegenerative Disease. GSE59685 and GSE134379 were also used to compare the DNAm ages of different tissues which were estimated by our trained cerebellum clocks. The DNAm samples of the non-cerebellar brain tissues in four datasets, i.e., GSE134379 [34], GSE74193 [40], GSE80970 [41], and GSE61431 [38], were used to train the CerebralCortexClock_common_ clock, and the four datasets were selected to ensure a relatively equal sample distribution across all age groups in the adult population.

### Data preprocessing

For all the DNAm datasets, after downloading from the GEO, they were read into R by using the *iadd2* function from the ‘bigmelon’ package [42] when raw IDAT files were available. For those datasets in which only text-formatted intensity files exist, the methylated and unmethylated intensities were extracted and read into R directly. Then, the raw methylation beta values are calculated as $\beta = \frac{M}{M + U + 100}$, where *M* denotes methylated intensities and *U* denotes unmethylated intensities. For all those samples, we estimated their sex by using the *estimateSex* function [43] from the wateRmelon package [44]; any samples with mismatches between its reported sex and the estimated sex from the DNAm data were excluded for downstream analysis. Also, the beta value density distributions of samples within each dataset were manually checked to remove any samples with abnormal distribution profiles.

### DNA methylation age prediction

The DNAm age prediction of the six representative clocks, i.e., Hannum2013 [3], Horvath2013 [4], Horvath2018 [30], Levine2018 [26], Zhang2019 [24], and Shireby2020 [16], was completed by using the *methyAge* function from the ‘dnaMethyAge’ R package [45]. Only methylation beta values are required to feed into the *methyAge* function. Note, when calculating the DNAm age of Horvath2013, the raw beta values are firstly normalized with an adjusted BMIQ which has a fixed reference; this is consistent with Horvath’s original publication [4]. To calculate the DNAm age of Zhang2019, the beta values of each sample are first subjected to Z-score normalization [24]. For the remaining clocks, no normalization steps were applied. The difference between DNAm age and chronological age is measured as follows:

1 $$\begin{aligned} RMSD=\sqrt{\frac{1}{m}\sum _{i=1}^{m}(y_i-\hat{y_i})^2} \end{aligned}$$

2 $$\begin{aligned} MAD=\frac{1}{m}\sum _{i=1}^{m}|y_i-\hat{y_i}| \end{aligned}$$

where $y_i$ represents the chronological age of the $i_{th}$ sample, $\hat{y_i}$ represents the predicted DNAm age of the $i_{th}$ sample, and *m* denotes the number of all samples. RMSD means root mean squared deviation; MAD means mean absolute deviation.

### Epigenome-wide association study

The age EWASs were performed on GSE134379 [34] which includes DNAm microarray data of two brain tissues (CBL and MTG) in every individual from a large elderly population (*N* = 404). The CBL samples and MTG samples were normalized by the *adjustedDasen* [46] from the ‘wateRmelon’ package [44] separately. These probes target CpGs mapped to sex chromosomes or reported to have cross-hybridizing issues and were removed from downstream analysis [47]. To find out age-associated differentially methylated CpGs across the genome in the two brain tissues, we fitted the following linear regression model for each CpG site involved in the two tissues separately:

3 $$\begin{aligned} \beta _{i} \sim w_{1i} * Age + w_{2i} *Sex + w_{3i} * Plate + w_{4i} * Beadchip + w_{5i} * Disease\_statue + intercept \end{aligned}$$

where $\beta _{i}$ is the methylation beta value of the $i_{th}$ CpG, and $w_{1i}$ is the coefficient of chronological age for the $i_{th}$ CpG. The *t statistic* of the coefficient $w_{1i}$ is checked in the *Student’s t* distribution to determine the *p*-value. After that, the *p*-values of all studied CpGs were adjusted with the Bonferoni correction method. A CpG is called to be significant age-associated when its adjusted *p*-value (or FDR) is less than 0.01.

### The construction of DNAm clocks

Prior to any training steps, all DNAm samples were normalized by a modified version of *adjustedDasen* [46] method from the ‘wateRmelon’ package [44], in which the modified *adjustedDasen* is supplied with a fixed reference to reduce the batch variance between different datasets. Also, the chronological age is log-transformed.

The new clocks mentioned in this study were all trained by the penalized linear regression algorithm—Elastic net [48], which is essentially a linear combination of the L1 and L2 penalties of the lasso regression and ridge regression. The loss function of Elastic net is defined as

4 $$\begin{aligned} \frac{1}{2} \sum w_i(y_i - \beta _i^T c - c_0)^2 + \frac{1}{2}\sum \lambda \gamma _j(1 - \alpha )c^2 + \alpha |c| \end{aligned}$$

where the $\beta _i$ denotes the methylation beta value of *ith* CpG, *c* is the coefficient vector of all the CpG accounted, and $\alpha$ is the critical parameter that controls the weights of the L1 and L2 penalties and has been defined prior to the training.

We used the *cv.glmnet* function from the ‘glmnet’ R package [49] to train the Elastic net models. To train the CerebellumClock_specific_, the input samples are the 752 cerebellum samples from six independent datasets, the input CpG set of each sample was restricted to the 613 age-associated CpGs in the cerebellum, alpha was set to 0.5, and 10-fold cross-validation was used to determine the optimal coefficient combination. We made use of leave-one(dataset)-out cross-validation to infer the age prediction performance of the CerebellumClock_specific_. Specifically, we have six independent cerebellum datasets, and then for each round of the total six rounds of cross-validation process, one dataset was taken out and their DNAm ages were estimated by the model trained on the remaining five datasets; after six rounds, the DNAm ages of samples from the six datasets were derived, and they were not overfitted by the training process. In the same way, the CerebellumClock_common_ was trained on the same 752 cerebellum samples, but the input CpG sets were restricted to the 201 shared age-associated CpGs. Another difference was that the alpha value was set to 0.2 to let the final model include more CpGs from the 201 CpGs.

**Fig. 1:**
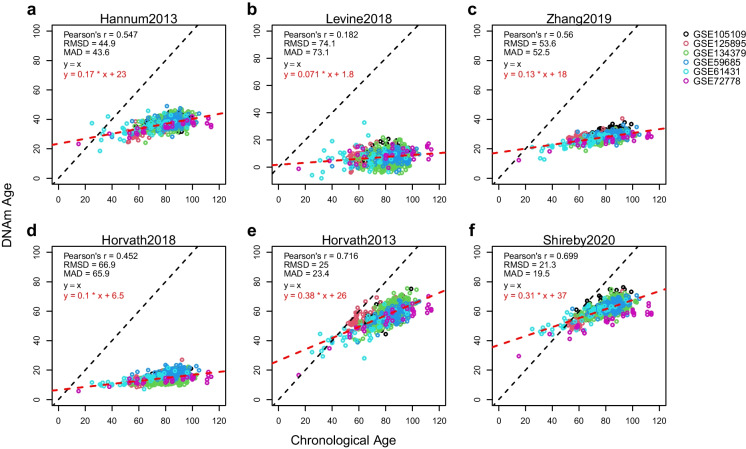
The cerebellum samples are severely underestimated by the six representative DNAm clocks. Each subplot illustrates results from different clocks: **a** Hannum2013, **b** Levine2018, **c** Zhang2019, **d** Horvath2018, **e** Horvath2013, and **f** Shireby2020. The colorful dots represent 752 cerebellum samples from six independent datasets, with different colors representing different datasets. The x-axis is chronological age, and the y-axis is the estimated DNAm age. The black dashed line represents the identical diagonal line between chronological age and DNAm age, and the red dashed line represents the regression line derived from regressing the DNAm age against the chronological age. RMSD, root mean squared deviation; MAD, mean absolute deviation

The training of CerebralCortexClock_common_ also employed Elastic net linear regression, the training samples were those of non-cerebellar brain tissues from four independent datasets, the input CpG set of each sample was also restricted to the 201 shared age-associated CpGs, and the alpha was set to 0.2. As we only have four separate datasets, and only GSE74193 [40] has a wide age range, we employed a 10-fold cross validation to measure the age prediction performance of CerebralCortexClock_common_. That is to say, we first randomly separate all the training samples into equal 10 portions; for each round of the total 10 rounds of cross-validation processes, we took one portion out, and their DNAm ages were then estimated by the model trained on the remaining 9 portions. After 10 rounds, the DNAm ages of samples from all ten portions were obtained, and they were not overfitted by the training process.

BrainCortexClock is trained on 640 cerebellum samples, which are from GSE134379 [34], GSE105109 [36], GSE125895 [37], GSE61431 [38], and GSE72778 [39], and 720 cerebral cortex samples, which are from GSE134379 [34], GSE61431 [38], GSE80970 [41], by Elastic net linear regression algorithm, and the input CpG sites were restricted to the 201 shared age-associated CpGs, and alpha was set to 0.2. As with the training of CerebralCortexClock_common_, ten-fold cross-validation was used to measure the age prediction performance of BrainCortexClock, and it was further tested by applying to an independent dataset of GSE59685 [35].

The coefficients of involved CpGs in each model are listed in Supplementary Tables 1.

### Software

All the analyses were conducted in R (version 3.6.0) [50] under a Linux environment. The scatter plots in Figs. 1, 3, and 4 were produced by the *getAccel* function with proper settings from the ‘dnaMethyAge’ R package [45]. The three constructed models of CerebellumClock_specific_, CerebellumClock_common_, and CerebralCortexClock_common_ are readily available to be applied in independent DNAm samples by calling the *methyAge* function from the ‘dnaMethyAge’ R package [45] with the ‘clock’ parameter setting as ‘Cerebellum_specific,’ ’Cerebellum_common,’ and ‘Cortex_common,’ respectively. GO analyses were conducted using the *gometh* function in the ‘missMethyl’ package [51] which tests gene ontology enrichment for significant CpGs while accounting for the differing number of probes per gene present on the 450k.

**Table 1:** Characteristics of the clean cerebellum samples from six datasets

**ID**	**Number**	**Female, Male**	**Age: mean (range)**	**Disease group**	**Reference**
GSE134379	404	200, 204	83.7 (54–103)	Alzheimer: 225 Normal: 179	[34]
GSE59685	111	64, 47	83.9 (40–105)	Alzheimer: 59 Normal: 52	[35]
GSE105109	95	41,54	81.2 (58–99)	Alzheimer: 67 Normal: 28	[36]
GSE125895	66	32, 34	67.3 (51.8${-}$92.3)	Alzheimer: 24Normal: 42	[37]
GSE61431	44	16, 28	61.6 (25–96)	Schizophrenia: 21 Normal: 23	[38]
GSE72778	32	21, 11	83.2 (15–114)	Alzheimer: 23 Normal: 9	[39]
Total	752	374, 378	80.63 (15–114)	Alzheimer: 398Schizophrenia: 21 Normal: 333	

## Results

### Characteristics of the DNAm cerebellum datasets

The cerebellum is a structure of the hindbrain, which plays a vital role in motor control [52]. Unlike peripheral tissues, such as blood or saliva, that can be non-invasively and repeatedly sampled, cerebellum samples are often collected from postmortem participants; as a result, there is a very limited number of DNAm cerebellum samples available. After rigorous searching on the Gene Expression Omnibus (GEO) database, where publicly available DNAm datasets are often deposited, we found a total of 6 datasets, each including more than ten cerebellum samples measured by Illumina 450k or EPIC array. After rigorous quality control (see ‘Methods’), 752 cerebellum samples remained and were used for downstream analysis. The biggest contributor for the final large cerebellum dataset is from GSE134379 [34], which contains 404 cerebellum samples. As cerebellum tissues were invasively collected from postmortem subjects, 90% of the collected samples were from individuals aged above 60 years old, with the median age at 80 years old. More detailed age, sex, and disease distribution information for each dataset is listed in Table 1. The DNAm microarray data from those datasets were originally produced to investigate disease-associated methylomic variations in the brain regions, especially for Alzheimer’s disease and Schizophrenia. As a result of this, our collected cerebellum samples include 333 samples with normal health status, 398 samples with Alzheimer’s disease, and 21 with Schizophrenia. The cerebellum is a relatively protected region, unlike other brain regions (such as prefrontal cortex); there generally are no significant AD-associated differences in the cerebellum [35, 37, 53]. Therefore, we included all these cerebellum samples, even those with disease diagnosis, for downstream analysis and also the following cerebellum DNAm age clock construction.

### Severe underestimation for cerebellum samples by various DNAm age clocks

Since 2013, many specialized and robust DNAm-based clocks have been reported. As recently suggested by Liu et al., those different clocks may have captured different biological processes of ageing considering their overall weak associations in the estimated DNAm age deviations [54]. Inspired by this, we investigated the DNAm ages of our collected 752 cerebellum samples predicted by six representative clocks: Hannum’s whole blood clock (Hannum2013) [3] and Horvath’s pan-tissue clock (Horvath2013)[4] are the two most widely used DNAm age clocks and especially Horvath2013 is reported to work well across many different tissue and cell types; Horvath’s blood &skin clock (Horvath2018) [30] is another multi-tissue clock and was reported to outperform Horvath2013 in epigenetic age prediction across several tissues; Levine’s PhenoAge clock (Levine2018) [26] was not directly regressing on chronological age and reported better prediction performance for all-cause mortality than other chronological age regressed clocks; Zhang’s blood clock (Zhang2019) [24] is reported the most accurate and robust age prediction model for blood samples; Shireby’s brain cortex clock (Shireby2020) [16] is a brain cortex specific clock and provides much better age predictions than other clocks in brain cortex tissues.

As shown in Fig. 1, almost all of the cerebellum samples are severely underestimated—they are all distributed below the diagonal lines. Hannum2013, Levine2018, and Zhang2019 are three age clocks trained almost exclusively on blood samples; the root-mean-square deviations (RMSDs) of their predictions are all very large (above 40 years), with Pearson correlations (*r*) ranging from 0.182 in Levine2018 and 0.56 in Zhang2019 (Fig. 1a–c); Horvath2018 is a multi-tissue clock that was trained on eight different tissues cell types but not including brain-related tissues; it produced a similar prediction trend (Fig. 1d) for cerebellum samples as the three blood clocks—large deviations (RMSD=66.9 years) and low correlation (*r*=0.452). In contrast, the underestimation effect is less apparent for Horvath2013 and Shireby2020 (Fig. 1e and f), their RMSDs are just above 20 years, and the Pearson correlation coefficient reached 0.699 by Shireby2020 and 0.694 by Horvath2013. We speculate the smaller underestimation effects by the two clocks are due to their training datasets having included a small ratio of cerebellum samples or entirely on brain cortex tissues. Specifically, Horvath2013 was trained on 3931 samples from 27 different tissues and cell types which include several different brain-related tissues including 282 cerebellum samples [4], while Shireby2020 was trained exclusively on brain cerebral cortices despite cerebellums not being involved [16]. It is worth noting that the regression lines of the estimated DNAm age against the chronological age by Horvath2013 and Shireby2020 both indicate that the cerebellum samples from young individuals aged below 30 years old are very likely to be overestimated (Fig. 1e and f).

### Smaller number of age-associated CpGs in the cerebellum methylome

We went further to investigate the underlying reasons why the cerebellum is systematically underestimated by the six age clocks. We hypothesized that, if the cerebellum truly ages slower than most other brain tissues, then due to a smaller ageing effect, there would be a much smaller number of CpGs passing the same cutoff to be identified as age-associated, and even those captured age-associated CpGs would mostly exhibit a smaller rate of methylation level changes with age. Inspired by this, we carried out two epigenome-wide association studies (EWAS) on age for the cerebellum (CBL) and the middle temporal gyrus (MTG) separately, based on the same dataset GSE134379 [34] which includes DNA methylation microarray samples of the two brain regions for every subject from a large elderly population (*n*=404).

**Fig. 2:**
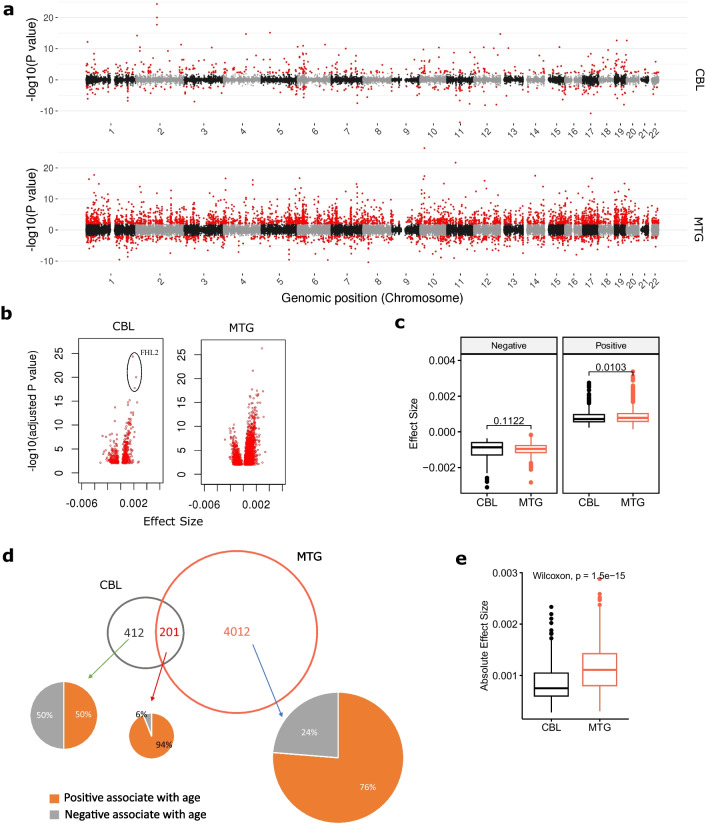
Comparison of age-associated methylation change between the cerebellum (CBL) and the middle temporal gyrus (MTG). **a** Manhattan plots illustrate the age EWASs results of CBL and MTG. Red dots denote significant age-associated CpGs (adjusted *p*-value $\le$ 0.01). **b** Two volcano plots show the effect size distribution of significant age-associated CpGs in CBL and MTG. **c** Boxplots comparing the age effect size in CBL and MTG for positive and negative age-associated CpGs, Wilcoxon Tests were performed, and Bonferroni-corrected *p*-values are displayed. **d** Venn plot shows the unique and shared number of the top 613 most significant age-associated CpGs in CBL and MTG. The three pie charts illustrate the proportions of CpGs gain methylation (positive associate with age) or lose methylation (negative associate with age) with age in three categories. **e** Boxplots comparing the absolute values of age effect sizes in CBL and MTG for the 201 shared age-associated CpG sites, Pairwise Wilcoxon Tests were performed, and Bonferroni-corrected *p*-value is displayed

We identified a total of 613 significant (Bonferroni-corrected *p*-value $\le$ 0.01) age-associated CpGs in CBL; in contrast, 4213 CpGs were found to be age-associated in MTG (Fig. 2a, b and Supplementary Tables 1). The top three age-associated CpGs in CBL are cg24079702, cg22454769, and cg06639320, which are all mapped to the *FHL2* gene, whereas the three loci exhibited similar age effect sizes though were less significant in MTG. When the age-associated CpGs in the two tissues were compared, only 32.8 % (201) of the CpGs in CBL were also identified as age-associated in MTG (Fig. 2d). More interestingly, when looking at the direction of ageing effect, CBL and MTG showed very different patterns in their age-associated CpGs. The CBL-only group has almost equal numbers of positive and negative age associations; in contrast, more than three-quarters (76%) of the MTG-only CpGs gain methylation with ageing. Moreover, the majority (94%) of the age-associated CpGs shared in the two tissues increase methylation levels with ageing (Fig. 2d); this is not very unexpected, as it has been shown that CpG sites exhibiting age-association in multiple tissues are more likely to gain methylation with age [14].

If the cerebellum ages slower, then it is reasonable to expect that the age-associated CpGs in the cerebellum would also have smaller ageing effect sizes; we then compared the ageing effect sizes of age-associated CpGs between CBL and MTG (Fig. 2b). Indeed, as shown in Fig. 2c, the ageing effect size of positive age-associated CpGs in CBL is generally smaller than that in the MTG (Wilcoxon test, Bonferroni-corrected $p=0.01$), though their difference is not significant in the negative age-associated CpGs ($p=0.11$). As regards the 201 shared age-associated CpGs (Fig. 2e), the difference in ageing effect size between CBL and MTG—smaller in CBL than MTG—is much more significant (Pairwise Wilcoxon test, Bonferroni-corrected $p<$1.5e$-$15).

Gene ontology analyses showed several enriched terms for the MTG-specific CpGs and Cerebellum-specific CpGs (Supplementary Tables 2) which included terms related to chromatin such as DNA binding, nucleosome assembly, and negative regulation of transcription by RNA polymerase II. The MTG-specific CpGs were enriched for pathways such as telomere organization, noradrenergic neuron differentiation, and dopaminergic neuron differentiation. Telomere shortening and neuron differentiation are both characteristics of cell mitotic divisions in cerebral cortex; thus, it suggests the MTG has a higher cell replication rate than cerebellum. In addition, the enriched GO terms for the cerebellum-specific CpGs were related to molecular functions such as DNA binding activity.

### Constructing DNAm age clocks for the cerebellum

#### Training the cerebellum specific DNAm clock

Our analyses in the previous section have clearly demonstrated that the six representative DNAm age clocks, including the pan-tissue clock and the cerebral cortex clock, all severely underestimated epigenetic ages of cerebellum samples. In addition, we have shown that the cerebellum has a much smaller number of age-associated CpGs. Then, we went further to find out whether it is possible to build an accurate age prediction model for the cerebellum.

**Fig. 3:**
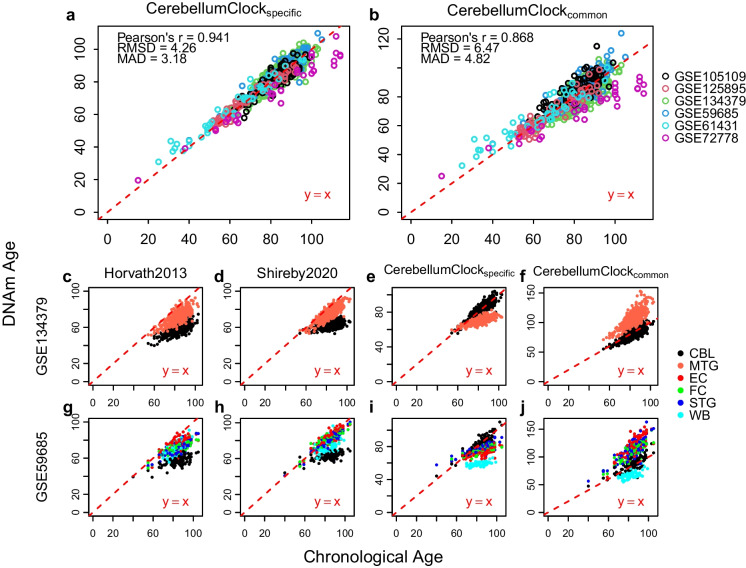
The cerebellum age clocks and their applications in other tissues. The leave-one(dataset)-out cross validation evaluates the age prediction performance of **a** CerebellumClock_specific_ and **b** CerebellumClock_common_ in cerebellum samples. Subplots **c**, **d**, **e**, and **f** compare the DNAm age of CBL and MTG estimated by Horvath2013, Shireby2020, CerebellumClock_specific_, and CerebellumClock_common_, respectively. Similarly, subplots **g**, **h**, **i**, and **j** compare the DNAm age of five different tissues estimated by the same four clocks. CBL cerebellum, MTG middle temporal gyrus, EC entorhinal cortex, FC frontal cortex, STG superior temporal gyrus, WB whole blood

We trained a cerebellum-specific age model, named CerebellumClock_specific_, by regressing the methylation beta values of the 613 age-associated CpGs from the 752 clean cerebellum samples against their corresponding chronological ages via the Elastic Net penalized linear regression algorithm [48]. The prediction performance of this model was measured by leave-one(dataset)-out cross validation (see ‘Methods’). As shown in Fig. 3a, the cross-validation results demonstrate that the trained cerebellum age models yield accurate age predictions for nearly all cerebellum datasets, except that most of the elderly subjects in GSE72778 were relatively underestimated. The overall Pearson correlation is above 0.94, with RMSD at 4.26 years and MAD is 3.18 years. The accurate age prediction performance of the cerebellum age model demonstrates that there is a persistent and significant ageing process undergoing in the cerebellum tissues.

#### Applying the cerebellum clocks in other tissues

To further examine the claim that cerebellum ages slower, we made another hypothesis: other tissues, including cerebral cortex and blood, would be significantly overestimated for their DNAm ages when measured by the cerebellum clock. To test this hypothesis, we then applied CerebellumClock_specific_ along with Horvath2013 and Shireby2020 in two separate datasets, GSE134379 and GSE59685, which both include cerebellum samples and samples of other tissues from the same subject. As expected, the cerebellum samples were apparently underestimated compared to other tissues by Horvath2013 and Shireby2020 in both GSE134379 and GSE59685 (Fig. 3). Interestingly, even though blood was also not included in the training set of Shireby2020, the predicated DNAm ages of blood samples in GSE59685 are still much higher than their counterparts in the cerebellum tissue (Fig. 3h).

However, when estimated by the cerebellum clock, the non-cerebellar samples were actually underestimated rather than overestimated compared to the cerebellum samples (Fig. 3i). This finding counters our previous expectation; we suggest that the underestimation effect for other tissues by the cerebellum clock may rather imply that this age model is working poorly in non-cerebellar tissues. In addition, we discovered that the cerebellum clock tends to overestimate the ages of non-cerebellar samples under 60 years old (Figs. 3e and i). This is further confirmed by looking at the overestimation facts for cortex tissues from young subjects by the cerebellum clock (Supplementary Figure 1). The penalized regression algorithm selected 275 age-associated CpGs from the 613 age-associated CpGs in the cerebellum, where the majority of them (73%) are on the CBL-only list, meaning they do not exhibit significant age correlations in MTG. Thus, the observed apparent underestimation effect for those non-cerebellar samples is not biologically meaningful, instead indicating artefacts resulting from improper usage of the age model CerebellumClock_specific_.

### Slower ageing rate in cerebellum according to two oppositely designed models

The above model CerebellumClock_specific_ thus captures cerebellum-specific age-related changes. In order to make more fair ageing rate comparisons, we then trained another cerebellum age model with the same regression algorithm and the same training samples, except the input CpG set is restricted to the 201 CpGs that are age-associated in both CBL and MTG. The leave-one(dataset)-out cross validation demonstrated that the new cerebellum clock, named CerebellumClock_common_, still gives very good age predictions for those cerebellum samples (Fig. 3b). Notably, CerebellumClock_common_ substantially overestimated the ages of brain cerebral cortices in both GSE134379 and GSE59685, though the ages of blood samples in GSE59685 were still underestimated (Figs. 3f and j).

**Fig. 4:**
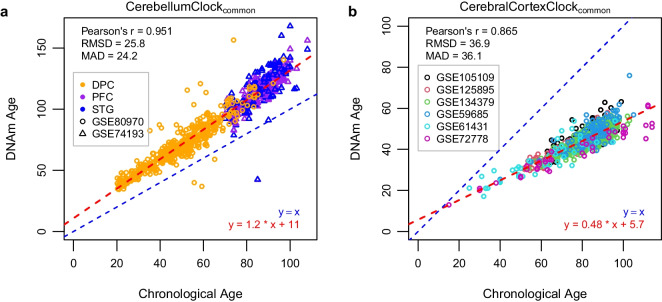
**a** The DNAm ages of samples of three different parts of human cerebral cortex (DPC dorsolateral prefrontal cortex, PC prefrontal cortex, STG superior temporal gyrus) in two datasets are systematically overestimated by CerebellumClock_common_. **b** The cerebellum samples are all severely underestimated by the CerebralCortexClock_common_. The CerebellumClock_common_ and CerebralCortexClock_common_ were trained from the same set of CpGs (*n*=201) but in different tissues. The blue dashed line represents the identical diagonal line between chronological age and DNAm age, and the red dashed line represents the regression line derived from regressing the DNAm age against the chronological age

To further confirm the overestimation effect for non-cerebellar brain tissues by the new cerebellum clock, we applied the CerebellumClock_common_ to two other independent datasets which, combined, include a large number of samples from three parts of cerebral cortex with a wide age range (20$\sim$100 years old). The results shown in Fig. 4a demonstrate that CerebellumClock_common_ substantially overestimates the whole age range of non-cerebellar brain tissues. The overall Pearson correlation coefficient reached 0.951, indicating the new cerebellum clock has also captured the strong ageing effect on the methylome of those tissues. More importantly, the slope of the regression line obtained from regressing the predicted DNAm age against the chronological age is greater than 1 (Slope $=1.2$), indicating that these non-cerebellar tissues have higher ageing tick rates than the cerebellum.

Likewise, we constructed another cerebral cortical clock, in which the training dataset includes samples from different parts of cerebral cortex, and the input CpG set was limited to 201 shared age-related CpGs. The resulting model, named CerebralCortexClock_common_ (Fig. 4b), performed well for samples from tissues that have been included in the training dataset (Supplementary Figure 2). We then applied it to the clean cerebellum dataset (*n*=752) we collected. As expected, all the cerebellum samples were largely underestimated by CerebralCortexClock_common_. Furthermore, the increasing deviations of the estimated DNAm ages from their chronological ages and the lower than 1 slope value of the regression line (Slope $=0.54$) indicate that the cerebellum ticks at a slower rate than other brain cortex tissues.

Altogether, we arrive at the same conclusion from the two oppositely directed analyses—the cerebellum has a smaller ageing tick rate when measured by models constructed by the same set of CpGs which were selected given they are age-associated in both CBL and MTG.

### Why does the cerebellum appear to age slowly

**Fig. 5:**
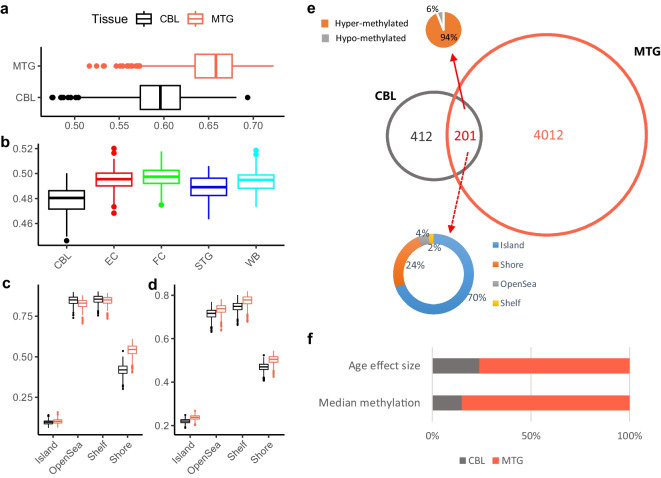
Boxplots illustrating the cerebellum (CBL) have a lower median overall methylation level than **a** MTG or **b** other four tissues (i.e., EC, FC, STG, and WB). **c** Median and **d** mean methylation comparison both agree the cerebellum has a lower methylation level in the CpG island and shore. Among the 201 common age-associated CpGs, 70% of them are located on the island, and 24% of them are located on the shore. The barplot in **f** shows the proportions of CpGs, which have larger age effect sizes or higher median methylation levels in CBL and MTG

We then sought to understand the underlying reasons why the cerebellum clock (CerebellumClock_common_) overestimated the ages of non-cerebellar brain tissues and the cerebral cortex clock (CerebralCortexClock_common_) underestimated the cerebellum tissue. Comparing the overall methylation levels, the cerebellum has an apparent lower median methylation level than MTG (Fig. 5a), and it also has the lowest median methylation level among the five tissue types included in GSE59685 (Fig. 5b). When grouping all CpGs into four genomic categories, i.e., island, open sea, shelf, and shore, the mean (Fig. 5c) and median (Fig. 5d) methylation comparison analysis both agreed that the cerebellum is less methylated in the island and the shore. Thus, it is reasonable to infer that the overall lower methylation level in the cerebellum mainly originated from its lower methylation level in the CpG island and the shore. It should be noted that we did not detect any significant correlations between mean methylation level change with age in any tissue types or the four genomic categories (Supplementary Figures 3 and 4), indicating the overall lower methylation level in the cerebellum is not due to a different ageing rate.

Next, we focused on the 201 common age-associated CpGs that were used to build CerebellumClock_common_ and CerebralCortexClock_common_. Firstly, we have shown that 94% of them gained methylation with age. Secondly, there are 140 CpGs on the island and 48 CpGs on the shore; they accounted for 93.5% ($p <2.2e-16$) of the 201 common CpGs (Fig. 5e). Consistent with that CBL has generally lower methylation levels in the CpG islands and shores than MTG, we found that the majority (85%, 171) of the common age-associated CpGs also have lower mean/median methylation levels in the cerebellum (Fig. 5f). Lastly, more than three-quarters of the 201 CpGs turned out to have a smaller ageing effect size in the CBL than MTG when regressing the methylation beta values against age, sex, and batches (Fig. 5f), meaning those CpGs have higher rates of age-associated methylation change in the MTG than the cerebellum. Altogether, the lower methylation levels, the positive age associations, and smaller ageing effect sizes of the majority of the common 201 CpGs in the cerebellum explain why CerebellumClock_common_ not only systematically overestimated the ages of non-cerebellar brain tissues (Intercept=11) but also with overestimation effect more prominent with age (Slope=1.2). Similarly, they also explain why the cerebellum samples were systematically underestimated by the CerebralCortexClock_common_.

**Fig. 6:**
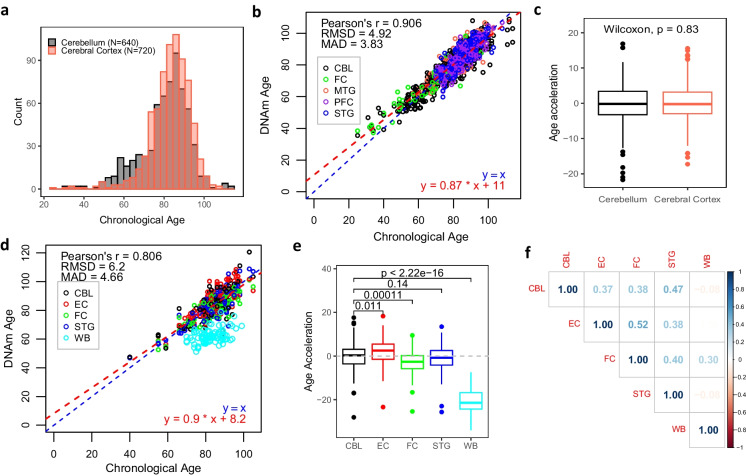
The clock of BrainCortexClock unbiasedly estimates DNAm age of cerebellum and cerebral cortex. **a** Age distributions of cerebellum samples and cerebral cortex samples in the training dataset. **b** Leave-one-fold-out cross-validation reveals the high performance of BrainCortexClock in training dataset. **c** Comparing age accelerations of samples from cerebellum and cerebral cortex in the training dataset demonstrates BrainCortexClock is not biased in the two tissues. The *p*-value was obtained from unpaired Wilcoxon Tests. **d** The performance of BrainCortexClock is evaluated in an independent dataset—GSE59685 which includes DNAm samples of five different tissues from 121 individuals. Note, the evaluation matrixes were drawn from samples that excluded whole blood. **e** Boxplots showing cerebellum samples are not systematically underestimated than three other parts of cerebral cortex; in contrast, whole blood is apparently underestimated. The *p*-values were obtained from pairwise Wilcoxon Tests. **f** Correlation matrix showing variations of age acceleration between cerebellum and other three parts of cerebral cortex were moderately correlated, and all four brain tissues were poorly correlated with whole blood. CBL cerebellum, MTG middle temporal gyrus, EC entorhinal cortex, FC frontal cortex, STG superior temporal gyrus, MTG middle temporal gyrus, PFC prefrontal cortex, WB whole blood

### A single clock unbiasedly estimates DNAm age of cerebellum and cerebral cortex

Though we have demonstrated that cerebellum shows different ageing patterns even on the shared 201 CpGs compared with cerebral cortex, the successful construction of CerebellumClock_common_ and CerebralCortexClock_common_ inspired us to investigate whether it is possible to build a single clock that works well, i.e., no systematic offset, for samples from both cerebral and cerebellar cortices. To start with, we selected a roughly equal number of cerebral cortex samples and ensured they have similar age distribution to the cerebellum samples (Fig. 6a). Then, the Elastic Net was applied to regress the methylation values of the 201 CpGs against the chronological age of samples from the two brain cortex tissues. Remarkably, the leave-one-fold-out cross-validation assessment showing the new brain cortex clock, named as BrainCortexClock, did accurately predict the age of samples from both cerebral and cerebellar cortices—the Pearson correlation coefficient reaches 0.906 and MAD is 3.83 years (Fig. 6b and c). We further tested its performance on an independent dataset—GSE59685 which includes DNAm samples from multiple tissues from 122 participants. Set aside the blood samples, the evaluation matrix generated from the brain cortex tissues further confirmed BrainCortexClock’s accurate age prediction performance (Fig. 6d). The boxplots in Fig. 6e demonstrate the cerebellum samples were not systematically underestimated and the cerebellum and cerebral cortex have similar levels of DNAm ages as estimated by this new clock; in contrast, the blood samples were apparently underestimated due to a lack of representation of this tissue in the training dataset. Furthermore, age acceleration comparisons between any two tissues from the same subjects showed the variations of age acceleration between cerebellum and other three parts of cerebral cortex were moderately correlated, with Pearson’s *r* ranging from 0.37 to 0.52 (Fig. 6f). Altogether, BrainCortexClock provides unbiased DNAm age prediction for brain cortex tissues including cerebellum.

## Discussion

In order to examine the claim that the cerebellum ages slower, we collected a large set of cerebellum samples (*N*=752) and assessed their DNAm ages from six representative clocks, including Horvath’s multi-tissue clock, i.e., Horvath2013. The results showed that these six representative clocks severely underestimated almost all cerebellum samples. This is consistent with previous reports [22, 32]. However, we should not conclude that the cerebellum ages slower only based on these results, as the underestimations may mainly reflect the improper usage of DNAm clocks, i.e., applying DNAm clocks in tissues which do not have adequate representations in the clocks’ training datasets. We found the underestimations were much more severe with the four clocks that were trained with no brain-related tissues—three clocks were trained mainly on blood tissues, and Horvath2018 was trained on eight other different tissues. In contrast, the underestimations were much attenuated in Shireby2020 whose training samples comprised non-cerebellar cortex tissues and Horvath2013 which included 282 cerebellum samples in its total 3931 training samples. Different tissues may have distinct DNA methylation profiles (particularly global methylation levels), and the dynamic changes of their methylomes in response to ageing also vary [14]. The Horvath’s multi-tissue clock produces relatively accurate age predictions for many vast different tissue/cell types [4]. Still, there is no evidence or guarantee to claim that it has captured the intrinsic mechanism that drives the DNAm changes across the whole body. We do not think it is justified to compare the ageing rates of different tissues by simply comparing their DNAm ages derived from the multi-tissue clock.

There exists a strong and consistent ageing effect on the DNA methylome of the cerebellum. By performing age EWAS on the cerebellum, we found 613 significantly age-associated CpGs from an elderly population; they were scattered across all autosomes. By taking advantage of penalized linear regression algorithm and a large training dataset, we constructed a highly accurate age clock for cerebellum (CerebellumClock_specific_, *r*=0.941, MAE=3.18 years). As a comparison, we identified many more age-associated CpG sites in a representative cerebral cortex tissue—MTG, and we found the CBL has smaller age effect sizes than the MTG, although it is only significant in the positive age-associated CpGs. We found 201 CpGs exhibiting age associations in both CBL and MTG; based on these 201 CpGs, we trained two clocks, i.e., CerebellumClock_common_ and CerebralCortexClock_common_, on all cerebellum samples and non-cerebellar cortex samples separately, they both performed well in age prediction for tissues that have included in their training dataset. When the two clocks are applied to samples from the cerebellum and non-cerebellar cortex tissues and the estimated DNAm ages are compared, they both agree that the cerebellum has a younger epigenetic age and a lower ageing rate. Furthermore, we have demonstrated that this is caused by 94% of the 201 CpGs gaining methylation with age, 85% are less methylated in CBL, and more than 75% have a smaller ageing effect size in CBL.

Even though our finding supports that the cerebellar methylome is more resistant to change with ageing, we should be cautious about whether this can be translated to the conclusion that the cerebellum is biologically younger than other human tissues. It should be noted that the above comparisons of ageing rates between cerebellum and MTG are based on the clocks trained on the same 201 age-associated CpGs. In fact, there is more than twice the number of CpGs found to be age-associated in the cerebellum and even more in MTG. When we apply the cerebellum-specific clock (CerebellumClock_specific_), which was trained by using all age-associated CpGs in the cerebellum, in predicting DNAm ages of other brain tissues, we could no longer observe a systematic overestimation for samples across all age groups; instead, only the individuals aged below 60 years old were overestimated; by contrast, the above 60 years old group was clearly underestimated (Fig. 3e, i and Supplementary Figure 1). We conclude that this is due to the improper usage of the clock, as the CerebellumClock_specific_ consists of many cerebellum-specific CpGs.

Why does the cerebellum have a much smaller number of age-associated CpGs? The observed age-associated methylation level change of CpGs sites in tissues with mixed cell types could arise through epigenetic drifts with mitotic divisions, cell type composition changes, and intrinsic changes affected by cell inner metabolism. Above 80% of cells in the cerebellar grey matter are non-replicating neuronal cells [55]. As a result of this, retrospective birth dating of cells through ^14^C bomb-pulse method indicates the average cell turnover rate in cerebellum is extremely low. In contrast, a much higher proportion of non-neuronal cells (mainly glial cells) in cerebral cortex makes it have a higher average cell turnover than the cerebellum [56]. *ELOVL2* hypermethylation has been demonstrated as a marker of cell divisions that occur throughout human ageing [57]; the hypermethylation of a locus in *ELOVL2*, which targeted by the probe of cg16867657, has been reported to show highest age correlation in whole blood [14, 58, 59]. Our results show that hypermethylation of cg16867657 is still significant (*p*=7.0e$-$08, effect size=0.0011) correlated with age in cerebellum though is much less significant than that in MTG (*p*=1.5e$-$12, effect size=0.0023), this is consistent with very low average mitotic rates in cerebellum. *FHL2* is another well-documented gene whose hypermethylation is strongly correlated with age [59–61]. Unlike *ELOVL2*, *FHL2* hypermethylation is not closely associated with cell replication [57]. Remarkably, the top three age-associated CpG sites in cerebellum are all mapped to *FHL2* gene, and they exhibited similar age effect sizes in MTG (Supplementary Tables 1), confirming hypermethylation of *FHL2* gene is not mainly accompanied by cell divisions. Taken together, we speculate that the smaller number of age-associated CpG sites found in cerebellum is largely attributed to its extremely low average cell replication rates.

It is easy to understand DNAm age comparisons between samples from the same tissues, i.e., we are confident that sample A is chronologically younger than sample B when the DNAm age of sample A is much smaller than sample B and they are from the same tissue. However, we still lack sufficient evidence to compare the biological ages of samples from different tissues confidently. For example, as recently reported by Jonkman and colleagues, Horvath’s multi-tissue clock predicts naive T cells to be up to 30 years younger than activated T cells from the same donor [62]. Can we conclude that naive T cells are biologically 30 years younger than activated T cells? Similarly, when predicted by our CerebellumClock_common_, the non-cerebellar brain tissues are predicted to be at least 11 years older than the cerebellum (Fig. 4a); however, we can not claim that those non-cerebellar brain tissues are biologically 11 years older than the cerebellum, as we could easily find one CpG or several CpGs combined that distinguishes the cerebellum from other brain tissue, then add it/them to the existing model and assigns it with a coefficient to counteract the 11 years gap. Then, the new adjusted clock should not produce DNAm age predictions with systematic large differences between the cerebellum and other brain tissues. As proposed by Liu et al., the many non-age-related CpGs in Horvath’s multi-tissue clock [4] may actually be reflecting and adjusting for tissue differences [54]. We have adequately demonstrated a single equation, BrainCortexClock, relying on only a subset of the 201 shared age-association CpGs provides unbiased DNAm age prediction for both cerebellum and cerebral cortex since given they have equal representation in the training dataset.

Another angle for ageing rate comparisons is to look at the Telomere Length (TL) shortening rates. Telomeres are protective DNA-protein complexes at the termini of chromosomes [63], and telomere attrition is considered an important hallmark of human ageing [1]. As comprehensively studied by Demanelis et al. [64], the average relative TL (RTL) varies across different tissue types; for instance, the average RTL is the lowest in whole blood and the longest in testis. Even though they found TL can shorten at different rates with ageing between several tissue types, the majority of tissues do not show a significant difference in age-dependent shortening rates, and there is no evidence to claim that different tissue types age at rates proportional to their TL shortening rates.

We should acknowledge some limitations of this study. First, due to the scarcity of cerebellum samples, the majority of our collected cerebellum samples are from elderly individuals aged above 60 years old. It would be very valuable to test our hypothesis that the Horvath’s multi-tissue clock would systematically overestimate the ages of cerebellum samples from young individuals aged below 30 years old. Second, our age EWASs on the cerebellum and MTG were also based on a very elderly population which has a relatively narrow age range; as demonstrated by Vershinina and colleagues [65], lots of age-associated CpGs do exhibit nonlinear methylation changes with age. Thus, our age EWASs may have missed many CpGs that are strongly age-associated in the younger age group but be a much-attenuated association in the aged group. Future studies that include more young individuals should reveal a more complete picture of age-associated changes in the cerebellar methylome.

Sugden et al. reported that approximately 77% of probes from the Illumina 450K array exhibit low test-retest reliability in blood, i.e., intraclass correlation < 0.4 [66]. When examining the CpGs utilized in constructing cerebellum clocks in this study, 130 out of the 201 overlapping age-associated CpGs fall into this category of poor reliability, accounting for 65% of them. Furthermore, within the CpGs employed by the BrainCortexClock and CerebellumClock_common_, 68% and 65%, respectively, are part of this group. Potential users applying these new cerebellum clocks to other datasets should be aware of this information.

## Conclusion

The large underestimations of age estimations for the cerebellum by widely used DNAm clocks are mainly due to inadequate cerebellum samples in their training datasets. We suggest the smaller number of age-associated CpG sites in cerebellum is largely attributed to its extremely low average cell replication rates. We have constructed a cerebellum-specific clock that can accurately predict cerebellum age and demonstrate conclusion from ageing rates comparison by DNA methylation clocks can be arbitrary by manipulating input CpG sites and the proportion of tissue types included in the training dataset. We believe our findings can have wider implications for the use of ageing clocks.

### Supplementary Information

Below is the link to the electronic supplementary material.

Supplementary file 1 (pdf 308 KB)

Supplementary file 2 (xlsx 490 KB)

Supplementary file 3 (xlsx 11 KB)
