# Supplementary material for: Insights into ageing rates comparison across tissues from recalibrating cerebellum DNA methylation clock
Source: GeroScience. 2023 Aug 19;46(1):39–56. doi: 10.1007/s11357-023-00871-w (PMC10828477; doi:10.1007/s11357-023-00871-w)
Supplement: Supplementary file 1 — (pdf 308 KB) [file 11357_2023_871_MOESM1_ESM.pdf]

Supplementary figures

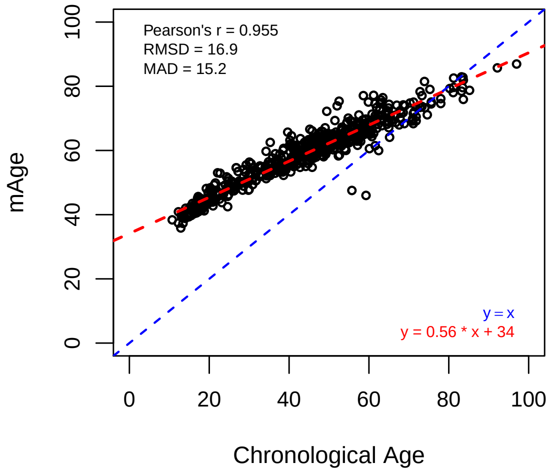

Supplementary Figure 1: Young cortex tissues are overestimated by CerebellumClock$_{\textbf{specific}}$. The cortex samples are from GSE74193.

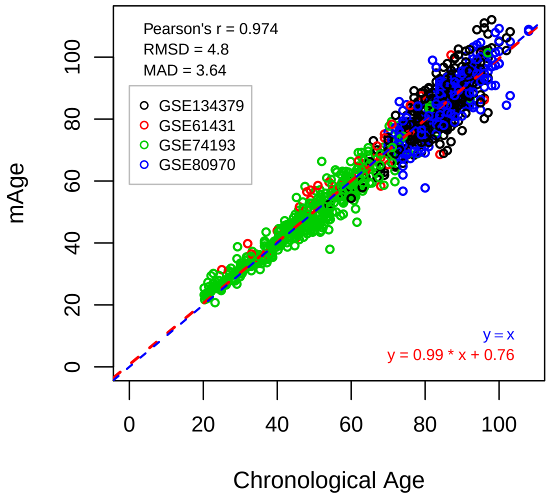

Supplementary Figure 2: The leave-one(fold)-out cross validation evaluate the age prediction performance of CortexClock$_{\textbf{common}}$. Colors represent samples from different datasets.

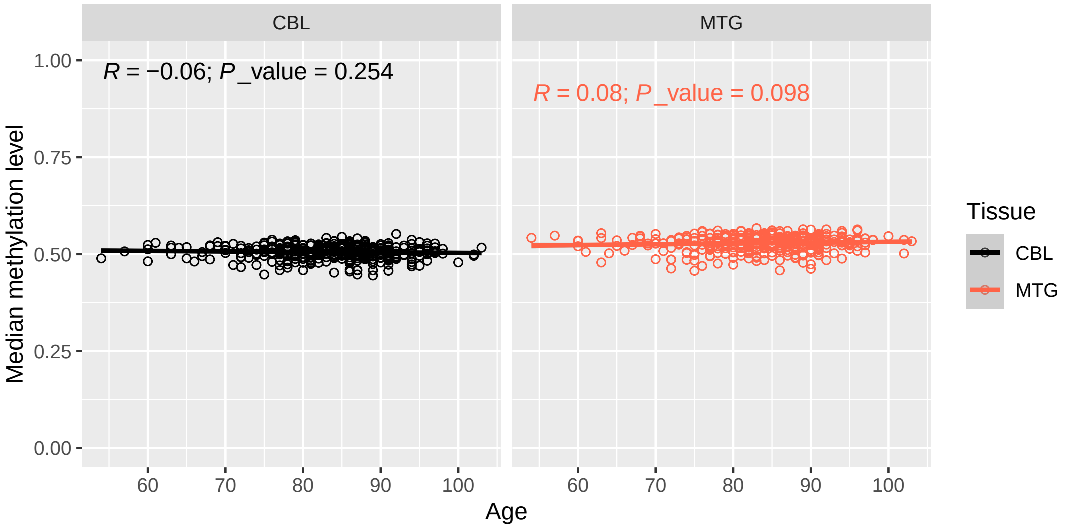

Supplementary Figure 3: The fluctuation of the mean methylation level is not correlated with chronological age either in the CBL or in the MTG.

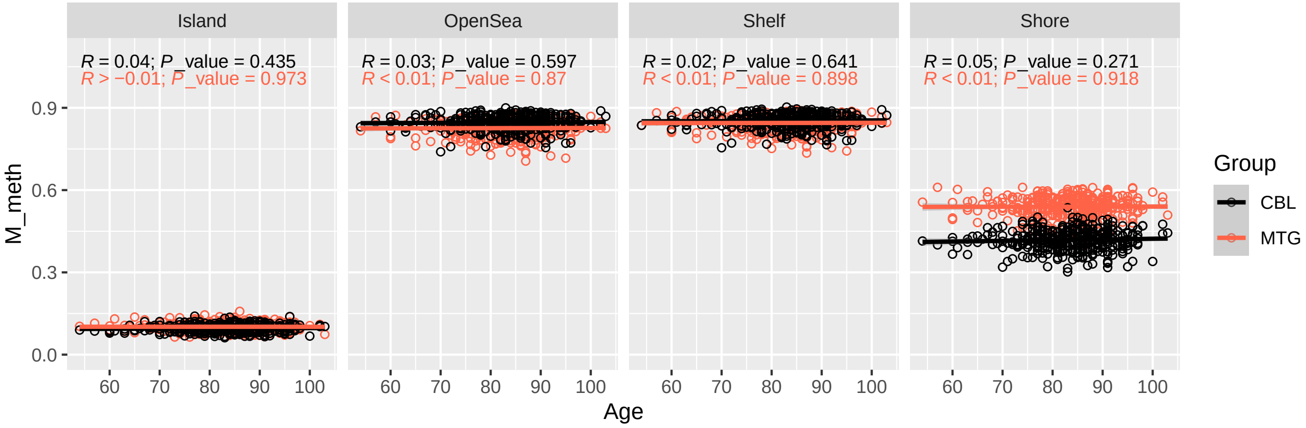

Supplementary Figure 4: The fluctuation of the mean methylation level is not correlated with chronological age in any of the four genomic regions, i.e. island, open sea, shelf and shore. Red denotes CBL sample and cyan denotes MTG sample.
